# Integrated Analysis of Parenchymal and Vascular HRCT Patterns with Circulating Biomarkers in Severe COVID-19 Pneumonia

**DOI:** 10.3390/diagnostics16040587

**Published:** 2026-02-15

**Authors:** Aldo Carnevale, Luca Morandi, Gaetano Scaramuzzo, Savino Spadaro, Gianluca Calogero Campo, Melchiore Giganti, Alberto Papi, Marco Contoli

**Affiliations:** 1 Radiology Section, Department of Translational Medicine, University of Ferrara, 44121 Ferrara, Italy; 2 Pneumology Unit, Cardiothoracic and Vascular Department, Sant’Anna University Hospital, 44123 Ferrara, Italy; luca.morandi@unife.it; 3 Intensive Care Unit, Department of Translational Medicine, University of Ferrara, 44121 Ferrara, Italy; gaetano.scaramuzzo@unife.it (G.S.);; 4 Cardiology Section, Department of Translational Medicine, University of Ferrara, 44121 Ferrara, Italy; 5 Pneumology Section, Department of Translational Medicine, University of Ferrara, 44121 Ferrara, Italy

**Keywords:** COVID-19, vascular signs, vascular tree-in-bud, ARDS, high resolution computed tomography

## Abstract

**Purpose**: To explore the correlation between radiologic patterns on high-resolution computed tomography (HRCT) and circulating biomarkers of inflammation and endothelial activation in patients with COVID-19 pneumonia, with the aim of identifying imaging-biomarker phenotypes that may offer insights for clinical stratification. **Materials and Methods**: This prospective single-center study included 84 consecutive patients hospitalized with PCR-confirmed SARS-CoV-2 infection and respiratory failure. All underwent baseline HRCT, along with parallel biohumoral profiling, including inflammatory (IL-1Ra, IL-6, IL-10) and endothelial (Angiopoietin-2, sVCAM-1, sE-Selectin) biomarkers. HRCT scans were reviewed for parenchymal and vascular abnormalities (vascular tree-in-bud [TIB], vascular enlargement pattern [VEP]). Semi-quantitative scores were assigned for parenchymal (PS) and vascular (VS) involvement. **Results**: Patients with higher PS had significantly prolonged hospital stay (35 vs. 17 days; *p* = 0.014), increased ICU admission rates (68.8% vs. 21.4%; *p* = 0.003), and elevated serum levels of IL-1Ra, IL-6, and IL-10 (*p* < 0.05). At multivariable analysis, PS remained independently associated with ICU admission after adjustment for age, inflammatory burden, and comorbidities (*p* = 0.014). A high VS was associated with significantly increased Angiopoietin-2 levels (*p* = 0.036), although it did not directly correlate with ICU admission or mortality. A significant positive correlation was observed between PS and VS (r =0.392; *p* < 0.001). **Conclusions**: in this study, HRCT-based parenchymal and vascular patterns appear significantly correlated with biological processes occurring in severe COVID-19 pneumonia. These observations, although preliminary, may offer a conceptual basis for future studies exploring radiologic and biomarker-based stratification in severe respiratory infections.

## 1. Introduction

The response of the medical and scientific communities to the COVID-19 pandemic has been unprecedented. In December 2019, a cluster of atypical pneumonia cases was noted by clinicians in Wuhan. Around this time, concern was growing in the global scientific community about a new outbreak of severe acute respiratory syndrome or a related disease, resulting in a global public health crisis of tremendous morbidity and causing 7 million global deaths as of 2024 [1].

Chest imaging plays a pivotal role in patients with COVID-19, narrowing the differential diagnosis, enabling early diagnosis, stratification of disease severity, identification of complications and potentially prognosticating such patients [2,3]. Notably, high-resolution computed tomography (HRCT) findings have shown to reflect pathologic changes beyond an anatomic resolution [4]. As a consequence of direct viral infection and indirect immunopathologic effects, both epithelial and endothelial lesions in COVID-19 occur simultaneously but independently. In the case of epithelial injury, the intact alveolar-vascular basement membrane is crucial in determining whether the organization caused by lung injury is resolved and reversible (typically represented as the organizing pneumonia pattern) or if lung injury continues to diffuse alveolar damage, clinically manifesting as acute respiratory distress syndrome (ARDS). On the other hand, a variety of CT findings can be identified in correlation with the various manifestations of COVID-19 pulmonary vasculopathy consequent to endothelial injury [4,5,6,7]. Moreover, it has been suggested that understanding different phenotypes of COVID-19 can lead to a personalized approach, which in turn may lead to better outcomes [8].

The world is still coping with the health, societal, and economic consequences of the COVID-19 pandemic. The understanding of exposure routes and pathogenic pathways for infectious respiratory diseases should always be among the first essential steps for scientists in response to future disease threats, because this knowledge should determine effective control strategies and focused therapeutic approaches with the aim of reducing morbidity and mortality. It would also be intriguing to evaluate whether the knowledge acquired during the COVID-19 emergency could be integrated into a more comprehensive framework for the management of respiratory tract infections in the post-pandemic phase. This would facilitate the decision-making process for physicians and, as a result, enhance the efficiency of healthcare resources [8,9,10,11,12].

In this work, we sought to investigate integrated radiologic, immunoinflammatory and endothelial injury biomarkers, and to determine whether radiological patterns of COVID-19 pneumonia, with a particular focus on vascular abnormalities, are useful in categorizing the patients in clinical-oriented subgroups and in establishing the prognosis.

## 2. Materials and Methods

This prospective, single-center study included consecutive patients admitted to the Respiratory and Intensive Care Units of the Azienda Ospedaliero-Universitaria di Ferrara (Ferrara, Italy) between 1 April 2020 and 28 February 2021.

This study builds upon and extends a previously published prospective investigation [13]. A subset of patients included in the current analysis had already been enrolled in the earlier cohort. However, the present study introduces an expanded dataset and incorporates novel radiological data and methodological approaches aimed at integrating laboratory biomarkers with high-resolution computed tomography (HRCT) features for improved clinical stratification.

Inclusion criteria were as follows:

- Confirmed SARS-CoV-2 infection by PCR-positive nasopharyngeal swab;
- Respiratory failure, defined as arterial oxygen tension (PaO_2_) < 8.0 kPa (60 mmHg) in room air and oxygen saturation < 90%;
- Availability of HRCT imaging at hospital admission.

Demographic, clinical, laboratory, and imaging data were collected through an anonymized electronic case report form (eCRF). All patients received standard-of-care treatment based on the best available evidence at the time.

### 2.1. Laboratory Procedures

Peripheral venous blood samples were obtained at admission (baseline), using a 21-gauge needle from an antecubital vein. Samples were collected early in the morning before administration of any pharmacological treatment.

Blood samples were analyzed for inflammatory cell counts as well as a comprehensive panel of biomarkers by multiplex immunoassay (EMD Millipore, Burlington, MA, USA), selected by an expert panel at our institution for research purposes, including:

- Endothelial activation markers: soluble E-Selectin (sE-Selectin, ng/mL), Angiopoietin-2 (ng/mL), soluble vascular cell adhesion molecule-1 (sVCAM-1, ng/mL), and soluble intercellular adhesion molecule-1 (sICAM-1, ng/mL);
- Inflammation and antiviral response markers: interferon-alpha 2 (IFN-α2, pg/mL), interferon-gamma (IFN-γ, pg/mL; t1), interleukin-1 receptor antagonist (IL-1Ra, pg/mL), interleukin-6 (IL-6, pg/mL), interleukin-10 (IL-10, pg/mL), interleukin-13 (IL-13, pg/mL), and soluble receptor for advanced glycation end products (sRAGE, pg/mL).

### 2.2. CT Data Acquisition and Quantification of Morphologic CT Abnormalities

CT scan acquisitions were conducted employing two different scanners: GE VCT Lightspeed 64 (GE Healthcare, Buckinghamshire, UK), Philips CT Brilliance 256 (Philips Medical Systems, Best, The Netherlands). The studies included a non-contrast acquisition and were obtained in the supine position from lung apices to bases at full-suspended inspiration, with ≤1.25 mm section thickness, using standard acquisition parameters adjusted to patients’ biometrics through automatic exposure control systems for dose optimization (100–120 effective mAs; 120–140 kVp). Images were reconstructed using a high spatial frequency kernel and visualized at window settings optimized for lung parenchyma (window level: −600 Hounsfield units (HU); window width: 1600 HU). All examinations were performed according to routine clinical practice in patients with moderate to severe COVID-19, in whom chest CT was considered clinically indicated in line with international recommendations.

CT features were interpreted by consensus of two radiologists blinded to clinical data (a thoracic radiologist with 7-year experience in chest imaging and a general radiologist with 6-year experience).

The overall extent of abnormal lung parenchyma was recorded, along with the sub-quantification of specific imaging patterns, including the percentage of ground-glass opacities (GGOs), crazy paving, and parenchymal consolidation. GGO was defined as a hazy increased attenuation area that does not completely obscure the underlying bronchi and vessels; crazy paving pattern was identified as septal thickening and intralobular reticular lines superimposed to GGO areas; consolidation was considered as an opacity causing complete obscuration of the underlying bronchial and vascular structures [14].

The semi-quantitative parenchymal CT score (PS; 0–5) was assigned based on the percentage of lung parenchyma affected by lung damage (1: <5%; 2: 5–25%; 3: 26–49%; 4: 50–75%; and 5: >75% involvement).

Vascular tree-in-bud (TIB) and vascular enlargement pattern (VEP), not obscured by dense parenchymal opacification, were identified.

**Figure 1:**
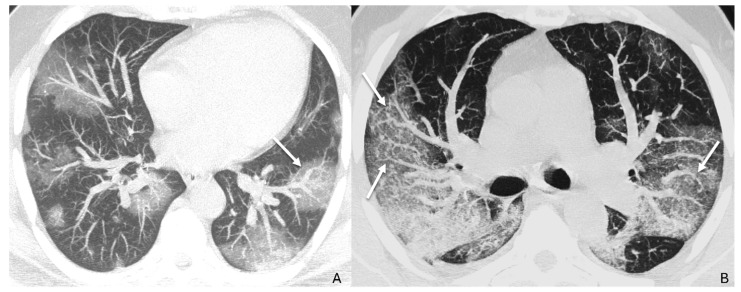
CT images, optimized lung window, MIP reconstruction. (**A**) Dilated segmental and subsegmental pulmonary vessels in the context of an area of ground glass (arrow); please make a comparison with comparable regions of non-diseased lung parenchyma. (**B**) Dilated and tortuous peripheral pulmonary vessels with a branching aspect resembling a budding tree and consistent with the vascular tree-in-bud pattern (arrows).

Vascular TIB was defined as the presence of dilated and tortuous peripheral pulmonary vessels with a branching aspect resembling a budding tree [5,15]. VEP was defined as a dilatation of segmental and subsegmental vessels with a diameter that exceeds the expected diameter for the point within the vascular tree [15]. This finding is characterized by: (a) a vessel diameter that is greater than that of adjacent portions of the non-diseased lung, (b) a vessel diameter that is greater than that of comparable regions of the non-diseased contralateral lung, or (c) focal dilatation or non-tapering of vessels as they progress toward the lung periphery (Figure 1).

Identification and evaluation of vascular abnormalities were aided by the use of maximum intensity projection (MIP) reconstructions, as necessary. The number of lobes demonstrating TIB and VEP was also separately recorded. For statistical analysis, a total vascular score was calculated as the sum of lobes displaying vascular TIB and/or VEP (VS; 0–12). At the time of study design, no validated or widely accepted vascular HRCT scoring systems were available for COVID-19 pneumonia. A simplified visual-based vascular score was therefore adopted as a pragmatic approach to summarize the burden of vascular abnormalities while limiting inter-observer variability.

### 2.3. Statistical Analysis

Descriptive statistics were used to summarize clinical, laboratory, and imaging characteristics.

For comparative analysis, the overall study population was stratified into two groups according to both the parenchymal and vascular CT scores using the median value of each score as the cut-off. Categorical variables were compared using Fisher’s exact test or Chi-square test, as appropriate. Continuous variables were analyzed using the Wilcoxon rank-sum test for unpaired comparisons, and the Kruskal–Wallis test followed by Dunn’s post hoc test or pairwise Wilcoxon tests for multiple comparisons.

Inter-reader reliability for VS and PS was assessed using the intraclass correlation coefficient (ICC), applying a two-way mixed-effects model with absolute agreement for single measurements.

In order to assess whether the parenchymal CT score (PS) was independently associated with ICU admission, a multivariable binary logistic regression analysis was performed. The PS was entered into the model as a continuous variable. The model was adjusted for age, C-reactive protein (CRP) levels as a marker of systemic inflammatory burden, and number of comorbidities at admission. Model fit was assessed using the chi-square test and Nagelkerke’s R^2^. Results are reported as odds ratios (ORs) with corresponding *p*-values.

All statistical analyses were performed using IBM SPSS Statistics, version 26.0 (IBM Corp., Armonk, NY, USA). A two-sided *p*-value < 0.05 was considered statistically significant.

## 3. Results

The total study cohort consisted of 84 patients fulfilling the inclusion criteria. The median age was 67 years (IQR 59–73), and 60 patients (71.4%) were male. Overall, in-hospital mortality in our population was elevated, with 27/84 patients deceased (32.1%).

Tables S1 and S2 summarize the baseline characteristics of the total study population, as well as subgroups stratified by parenchymal and vascular CT scores. No significant differences were identified in the distribution of gender or comorbidities such as asthma, COPD, ILD and cancer among the high and low PS and VS groups.

Inter-reader agreement for the assessment of VS was excellent, with an ICC of 0.92 (95% CI: 0.87–0.95; *p* < 0.001); for PS, instead, inter-reader agreement was good-to-excellent, with an ICC of 0.89 (95% CI: 0.83–0.92; *p* < 0.001).

### 3.1. Parenchymal CT Score

Patients with a higher PS showed significantly lower hemoglobin (Hb) levels compared to those with a low score (11.45 vs. 12.15 g/dL; *p* = 0.041), suggesting a possible association between anemia and more extensive parenchymal involvement.

Moreover, hospitalization parameters differed significantly between the two groups. Patients with high PS experienced a significantly longer duration of hospital stay (35 vs. 17 days; *p* = 0.014), and were more frequently admitted to the intensive care unit (ICU) where they experienced significantly longer stays. Specifically, 68.8% of ICU-transferred patients had a high PS, compared to 21.4% in the low PS group, with a median duration of ICU stay of 25 vs. 11 days (*p* = 0.003).

Regarding inflammatory and immunological profiles, higher parenchymal involvement was associated with increased levels of:

- Interleukin-1 Receptor Antagonist (IL-1Ra) (*p* = 0.017);
- Interleukin-6 (IL-6) (*p* = 0.014);
- Interleukin-10 (IL-10) (*p* = 0.045).

These findings support the hypothesis that elevated parenchymal burden in COVID-19 is linked not only to greater clinical severity, but also to enhanced systemic inflammatory response and immune dysregulation.

**Figure 2:**
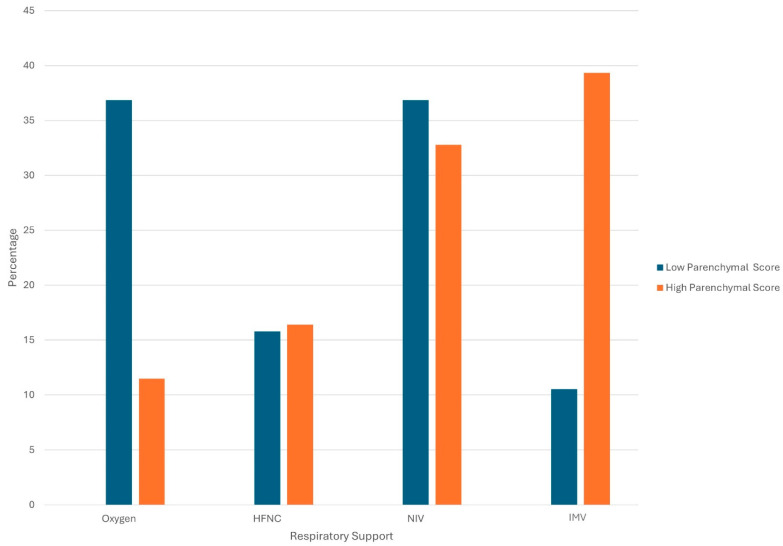
Distribution of respiratory support modalities according to parenchymal HRCT score. Histogram showing the percentage of patients requiring different levels of respiratory support—conventional oxygen therapy, High-Flow Nasal Cannula (HFNC), Non-Invasive Ventilation (NIV), and Invasive Mechanical Ventilation (IMV)—stratified by low vs. high parenchymal involvement on HRCT. Patients with higher parenchymal scores were more likely to require advanced ventilatory support (NIV or IMV).

The type of respiratory support at admission was significantly associated with parenchymal involvement (Pearson’s χ^2^ = 9.103, *p* = 0.028). In particular, invasive mechanical ventilation was more frequently required in patients with a higher parenchymal score (39.3% vs. 10.5% in the low-score group; Figure 2).

To further explore the relationship between parenchymal involvement and clinical severity, a multivariable binary logistic regression analysis was performed with ICU admission as the dependent variable. The overall model was statistically significant (χ^2^ = 24.64, df = 4, *p* < 0.001), with a Nagelkerke R^2^ of 0.587. After adjustment for age, CRP levels, and number of comorbidities, the PS remained independently associated with ICU admission (OR 2.57 per unit increase, *p* = 0.014), supporting its role as an independent predictor rather than a mere proxy of overall disease severity. Higher CRP levels were also independently associated with ICU admission (OR 1.21, *p* = 0.006), whereas age and number of comorbidities were not significantly associated with the outcome. The model demonstrated good discriminative performance, with an overall classification accuracy of 81.4%.

### 3.2. Vascular CT Score

Among the analyzed parameters, Angiopoietin-2 levels were significantly elevated in patients with a high VS (*p* = 0.036), supporting the role of endothelial activation and vascular injury in the pathophysiology of COVID-19 pneumonia.

**Figure 3:**
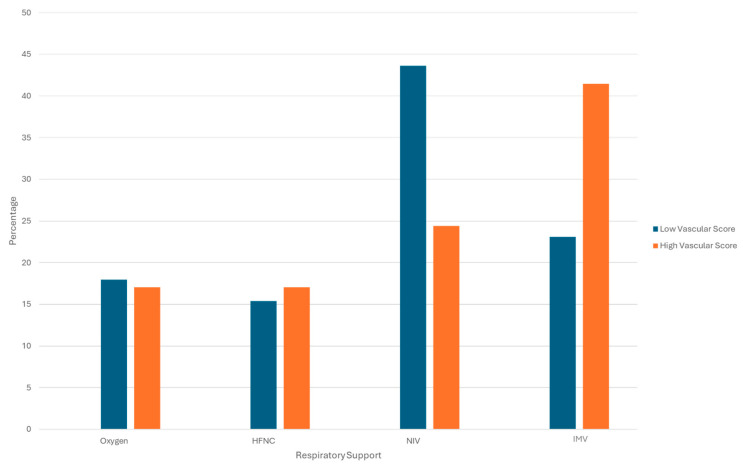
Distribution of respiratory support modalities according to vascular HRCT score. Histogram showing the percentage of patients requiring different levels of respiratory support—conventional oxygen therapy, High-Flow Nasal Cannula (HFNC), Non-Invasive Ventilation (NIV), and Invasive Mechanical Ventilation (IMV)—stratified by low vs. high vascular score on HRCT. The distribution of respiratory support modalities did not differ significantly between groups.

No statistically significant differences were observed for other biohumoral or clinical variables between VS categories (Figure 3).

Interestingly, a significant positive correlation was observed between parenchymal and vascular involvement (Spearman’s rho = 0.392, *p* < 0.001). This suggests a pathophysiological link between microvascular damage and the extent of alveolar inflammation, although the two processes may manifest with different degrees of severity in individual patients.

## 4. Discussion

In this study, we hypothesized correlations between HRCT features and circulating biomarkers reflecting immunoinflammatory and endothelial damage pathways in hospitalized patients with severe COVID-19.

Higher PS was significantly associated with elevated levels of IL-1Ra, IL-6, and IL-10, as well as markers of clinical severity, including longer hospitalization, ICU admission, and length of ICU stay along with the need for invasive ventilation. Importantly, multivariable analysis demonstrated that the degree of parenchymal involvement remained independently associated with ICU admission after adjustment for age, systemic inflammatory burden (CRP), and number of comorbidities, supporting its role as an independent imaging biomarker of disease severity rather than a mere surrogate of inflammation.

Such data are consistent with the concept that the extent of lung parenchymal damage mirrors systemic inflammation and more severe disease presentation, as reflected by the need for advanced respiratory support. Higher VS significantly correlated with increased levels of Angiopoietin-2, thus confirming its relationship with pronounced endothelial activation. However, the VS failed to demonstrate a correlation to specific clinical features, apart from the biohumoral data.

Since the early stage of the pandemic, radiologists have promptly responded to COVID-19 by identifying the CT findings of the disease, which include GGOs with or without consolidation, GGOs with intralobular lines (i.e., a “crazy paving” appearance), and other findings of organizing pneumonia in peripheral and bilateral distributions, with a typical temporal evolution [2]. The use of CT scans as a first-line tool for COVID-19 diagnosis has been discouraged by guidelines [3,16], as the typical CT findings of the disease may overlap with those of differential diagnoses (including cryptogenic organizing pneumonia or influenza pneumonia). However, chest CT was crucial in the management of cases that were challenging to diagnose solely on the basis of a polymerase chain reaction test [12,17]. Chest imaging is also beneficial in the detection of COVID-19-associated complications, including pulmonary thromboembolism, superimposed pneumonia other than COVID-19, and heart failure, particularly when patients experience abrupt deterioration of clinical signs or symptoms. Approximately 20% to 30% of individuals infected with SARS-CoV-2 develop a moderate-to-severe disease, with a substantial proportion progressing to acute respiratory distress syndrome (ARDS), leading to ICU admission and associated with great mortality, approaching 30% [18]. In these patients, thoracic imaging typically reveals widespread GGOs and areas of consolidation. It has been reported that COVID-19-related ARDS differs from non-COVID-19 ARDS with respect to several radiological and physiological features [19,20,21]. Indeed, the severe form of COVID-19 is now recognized as a syndrome characterized not only by profound inflammation and lung epithelial injury but also by endothelial dysfunction and coagulopathy. The elevated mortality in this group may also be attributable to an uncontrolled interplay between immune and thrombotic mechanisms—a phenomenon commonly described as “immunothrombosis” [4,6,13,22]. The radiological severity—quantified by HRCT patterns such as GGOs, consolidations, and vascular involvement—has been shown to correlate with histopathologic findings including diffuse alveolar damage, endothelialitis, and microvascular thrombosis [4,6,23]. Doglioni and colleagues confirmed in vivo this correlation using lung cryobiopsy specimens obtained in the acute phase of the disease [24].

Our data, although exploratory, suggest that HRCT may help distinguish a low-parenchymal involvement phenotype, with less impact on respiratory function and possibly better outcomes, from a high-parenchymal involvement phenotype associated with a worse clinical course. Conversely, the lack of an association between the VS and clinical deterioration is more difficult to interpret. It may indicate that radiologically documented vascular enlargement and ‘tree-in-bud’ patterns do not directly drive the severity of acute respiratory failure, but rather represent imaging correlates of underlying endothelial and microvascular alterations. It has to be considered, nonetheless, that a significantly positive correlation was observed between PS and VS, suggesting the existence of a phenotype in which both microvascular damage and alveolar inflammation coexist, characterizing the most severe patients. The good to excellent inter-observer agreement we identified, along with the high availability of CT scan evaluation in the context of ARDS, makes this semi-quantitative evaluation promising. At present, these observations should be interpreted as exploratory and descriptive, and do not support clinical decision-making or risk stratification. However, if confirmed in larger cohorts, combined assessment of lung damage and vascular injury may contribute to a more nuanced description of disease heterogeneity in severe COVID-19, with possible prognostic and predictive implications.

As previously mentioned, the VS is limited by the potential underestimation of vascular signs, which can be masked by consolidation and/or atelectasis frequently occurring in the later phases of the disease. This may partly explain the lack of correlation between VS and clinical outcomes observed in our study. More advanced techniques, such as dual-energy CT, may help obtain a more precise definition of vascular involvement thanks to the ability to depict directly lung parenchymal perfusion.

The role for biomarkers in COVID-19 pathophysiology has been explored by other groups. The correlation between the CT appearance of COVID-19 pneumonia and laboratory biomarkers, however, has not been thoroughly investigated so far, typically representing a secondary outcome in retrospective studies. For instance, a study by Herold et al. [25] demonstrated that IL-6 and CRP levels may serve as predictors of patients in need of ventilator support. However, patients requiring mechanical ventilation did not differ in terms of radiological findings.

D’Agostini and colleagues [26], investigating the role of mid-regional pro-adrenomedullin in risk stratification in COVID-19 patients upon admission to the emergency department, found that the CT score correlated with all the biomarkers considered (including CRP, procalcitonin, lactate dehydrogenase and D-dimer), and showed a significant, although unsatisfactory, discriminative performance both for in-hospital mortality and for mechanical ventilation.

In our study, a higher PS was significantly associated with elevated serum levels of IL-1Ra, IL-6, and IL-10 and worse clinical severity indicators. This observation is consistent with the growing body of evidence indicating that the extent of lung parenchymal involvement in COVID-19 reflects not only direct virus-induced injury, but also the degree of systemic immune response. In a previous study on moderate/severe COVID-19 cohort of patients in our institution, Contoli et al. [13] demonstrated that patients with more severe disease exhibit a dysregulated cytokine profile characterized by simultaneous elevation of pro-inflammatory (IL-6) and regulatory/anti-inflammatory (IL-1Ra, IL-10) mediators. The authors suggested a state of maladaptive inflammation associated with an ineffective innate immunity, proved by deficient IFN-alpha production in deceased patients as compared to the survived ones. IL-6 plays a central role in promoting endothelial activation, vascular permeability, and systemic hyperinflammation, and its levels have been associated with worse clinical outcomes in non-COVID-19 ARDS [27]. In a large cohort of patients from the New York area, Del Valle et al. also identified higher levels of IL-6 to correlate with a more severe form of the disease and with worse outcomes [28]. Accordingly, IL-6 is now recognized as a major marker of severe COVID-19 disease, with a predictive role and therapeutic implications [29,30,31].

The role of IL-1ra and IL-10, however, is less clear. IL-1Ra, though classically considered an anti-inflammatory molecule, is markedly upregulated in severe cases, possibly reflecting compensatory immune activation in the face of unchecked IL-1 signaling. IL-10, similarly, may serve both protective and pathological roles, acting as an anti-inflammatory brake that paradoxically coexists with hyperinflammatory processes in critical illness [13]. The association of these cytokines with higher PS supports the hypothesis that the radiological extent of lung injury is tightly linked to immunological derangement. Indeed, the radiological severity—quantified by HRCT patterns such as GGOs, consolidations, and vascular involvement—correlates with histopathologic findings including diffuse alveolar damage, endothelialitis, and microvascular thrombosis [4,6,23]. Therefore, the degree of parenchymal involvement on HRCT may serve as a surrogate marker for systemic inflammation and immune-mediated organ damage. However, the practical clinical utility of combining imaging with biomarker profiling requires further validation in larger, longitudinal cohorts before it can be recommended to guide immunomodulatory interventions.

A distinct vascular phenotype has also been described in COVID-19. Imaging and pathological studies have shown that this phenotype includes features such as pulmonary vessel enlargement, impaired hypoxic vasoconstriction, microangiopathy, and intrapulmonary shunting, contributing to severe hypoxemia disproportionate to the degree of parenchymal involvement [6,32,33]. Microvascular alterations, including endothelialitis, micro-thrombosis and neo-angiogenesis, have been shown in pathological samples from deceased patients with COVID-19-related ARDS by Ackelmann et al. [34]. These vascular changes, driven by a combination of viral endothelial tropism and inflammatory cytokine-mediated injury, support the integration of vascular imaging biomarkers into clinical stratification models.

Vascular involvement has emerged as a critical hallmark of COVID-19 pneumonia, with HRCT revealing a range of microvascular abnormalities [4,5,15,35,36,37]. Among these, peripheral vascular enlargement and the vascular tree-in-bud (TIB) pattern have been recognized as distinctive imaging features. The morphology and peripheral distribution of the dilated, branching vessels observed in some patients resemble those described in rare cases of pulmonary tumor thrombotic microangiopathy, suggesting a shared microangiopathic substrate. Microvascular alterations such as vascular enlargement and TIB are now considered part of the typical radiological spectrum of COVID-19; however, their diagnostic and prognostic significance remains incompletely understood. In a seminal work, Patel et al. [5] first documented microvascular abnormalities on CT in patients with severe COVID-19, proposing that the vascular TIB appearance may reflect underlying processes of immunothrombosis and pathological angiogenesis. Supporting this hypothesis, Şanlı et al. [33] reported that peripheral vascular changes—including vessel irregularity and tapering—correlated with markers of systemic inflammation such as C-reactive protein (CRP), lactate dehydrogenase (LDH), and clinical symptoms like dyspnea.

A study by Dalpiaz et al. [15] reported that the presence of vascular enlargement and TIB patterns involving three or more pulmonary lobes was more commonly observed in patients who showed poor response to prone positioning. However, no significant association emerged between these radiologic findings and key clinical outcomes, including duration of mechanical ventilation, mortality, or median levels of biomarkers linked to disease severity and thrombotic activity. This discrepancy across studies highlights that, although HRCT can identify localized vascular anomalies, these do not necessarily translate into measurable systemic or clinical deterioration in every cohort.

In this scenario, our findings reinforce the role of endothelial activation in the pathophysiology of COVID-19-related lung injury in severe cases. Among the analyzed biomarkers, only Angiopoietin-2 levels were significantly elevated in patients with high vascular HRCT scores (*p* = 0.036), supporting its value as a marker of endothelial dysfunction and vascular damage. Interestingly, no significant differences were found in other biohumoral or clinical parameters between vascular score categories, underscoring the specificity of Angiopoietin-2 as a correlate of radiologic vascular injury. Moreover, we observed a strong association between vascular and parenchymal involvement: patients with high vascular scores also showed significantly higher parenchymal scores (*p* = 0.002), suggesting a pathophysiological link between microvascular damage and the extent of alveolar inflammation and consolidation. This overlap likely reflects the dual mechanism of COVID-19 lung injury, characterized by both inflammatory and thrombotic processes that affect the alveolar-capillary unit. Vascular enlargement and peripheral vessel pruning on HRCT are frequent in COVID-19 and may represent an imaging surrogate of microvascular occlusion and perfusion imbalance [4,38].

The elevation of Angiopoietin-2 in COVID-19 has been consistently linked to respiratory deterioration, increased need for mechanical ventilation, and adverse clinical outcomes, underscoring its role as a biomarker of endothelial dysfunction and disease severity [34,39,40]. Histopathological studies have provided mechanistic insight into these associations: Ackermann and colleagues [34] identified widespread endothelialitis, microthrombosis, and pronounced intussusceptive angiogenesis in post-mortem lung specimens from COVID-19 patients, distinguishing this disease from classical ARDS and pointing to a distinct vasculopathic phenotype. Further works demonstrated that circulating markers of endothelial activation, including Angiopoietin-2, strongly correlate with COVID-19 severity, ICU admission and mortality, suggesting a systemic endothelial response to viral injury and inflammatory cytokines with prognostic impact [39,40,41].

Leisman and colleagues analyzed more than 200 patients enrolled at the ER department with COVID-19 requiring respiratory support and longitudinally measured a set of biomarkers looking for correlations with clinical outcomes. The authors found an early peak in lung epithelial damage markers such as RAGEs with a subsequent drop, followed by a later peak in endothelial activation markers including Angiopoietin-2, which also correlated with a more severe clinical course and worse outcome [42]. A similar correlation was also identified by Spadaro et al. in a cohort of ICU-admitted and mechanically ventilated patients, differentiating COVID-related ARDS from other ARDS forms [43]. It must be noticed, however, that conflicting results exist. Bhatraju et al. found lower levels of Angiopoietin-2 among ICU critically ill COVID-19 patients as compared to a control group of non-COVID-19 critically ill ICU patients admitted in the same time frame. The authors also found no correlation between Angiopoietin-2 levels and death [44].

We did not observe a significant correlation between the vascular imaging score and clinical outcomes such as ICU admission, need for mechanical ventilation, or in-hospital mortality. This discrepancy suggests that radiologically evident vascular involvement may not directly reflect the systemic vascular burden or the degree of physiological impairment in all patients. One possible explanation lies in the temporal dynamics of the disease: HRCT was performed at a single time point, which may not capture the full evolution of microvascular injury or its clinical consequences. This is in line with the time frame presented by Leisman et al. [42]. Considering our enrolment time frame, occurring in between emergency room presentation and mechanical ventilation/ICU admission, it most likely corresponds to the period between the peak of epithelial damage and the peak in endothelial marker release, which may have influenced the correlations in our analysis. Moreover, radiological signs such as vascular enlargement and TIB pattern likely reflect localized endothelial dysfunction or perfusion anomalies, whereas adverse outcomes may depend on a more diffuse or systemic endothelial activation that is not fully detectable by imaging. The previously mentioned limits of VS may also account for these findings.

The present study has some limitations. First, the limited sample size, linked to the monocentric design and to the strict inclusion criteria applied during the pre-vaccination phase of the COVID-19 pandemic, may have reduced the statistical power of the analyses. Notably, the cross-sectional design of the study precludes any causal inference, and observed associations should be interpreted within a descriptive and hypothesis-generating framework. Also, its monocentric nature may limit the generalizability of the findings to other populations. Second, a control group consisting of patients with ARDS due to other conditions (e.g., non-COVID-19 ARDS or non-infectious severe hypoxemia) was not included, preventing a broader comparison across etiologies. This may limit the ability to determine whether the observed imaging–biomarker associations are specific to COVID-19 or rather reflect more general features of severe respiratory failure. Third, the timing of inclusion may have varied in relation to disease onset, potentially introducing heterogeneity in the interpretation of both clinical and imaging findings. Finally, the CT score has its own limits, as previously discussed in this manuscript, notably potential underestimation of vascular involvement on CT imaging due to superimposed atelectasis or alveolar opacification. Advanced quantitative imaging approaches and artificial intelligence-based analysis may help overcome some of the intrinsic constraints of visual-based assessment, enabling a more objective and reproducible characterization of vascular abnormalities, particularly in the presence of extensive parenchymal disease [45,46,47].

Notwithstanding these limitations, the study presents several strengths. It was conducted prospectively, with consecutive patient enrolment and predefined severity criteria, thereby reducing selection bias and enhancing the internal validity of the results. The availability of both imaging and biohumoral assessments provides insights in a poorly explored field, where current knowledge is largely based on retrospective, post-mortem, or ex vivo analyses. The accurate collection of clinical data allows for exploratory speculations on patient stratification with prognostic and possibly predictive implications. Our study is in line with the most recent discussion on phenotyping ARDS, and despite the lack of control groups, it confirms previously reported observations on biomarkers in severe SARS-CoV-2 with its peculiar vascular damage in the lung when compared to other forms of ARDS. Finally, given that the national vaccination campaign in Italy began in January 2021 with healthcare workers, the cohort exclusively comprises unvaccinated individuals, thus reflecting an immunologically naïve host–virus interaction. On the other hand, the present findings may not be generalizable to vaccinated populations, and this distinction warrants further investigation in future studies.

## 5. Conclusions

Our study provides novel, albeit preliminary, insights into the relationship between radiological patterns and biochemical markers in severe COVID-19 pneumonia. Specifically, patients with higher parenchymal involvement scores exhibited significantly increased levels of inflammatory cytokines, including IL-1Ra, IL-6, and IL-10, and experienced longer hospital stays, higher rates of ICU admission, and a greater need for invasive mechanical ventilation. Notably, the parenchymal score remained independently associated with ICU admission after adjustment for age, inflammatory burden, and comorbidities, reinforcing its potential value as an independent imaging predictor of clinical deterioration. These findings support a close association between extensive parenchymal lung involvement, disease severity, and systemic immune dysregulation.

Notably, Angiopoietin-2 levels were selectively associated with vascular abnormalities on HRCT, reinforcing the role of endothelial activation in the disease pathogenesis. In contrast, we found no direct association between vascular imaging patterns and clinical outcomes, highlighting the complexity of vascular injury and the need for integrative diagnostic approaches.

Importantly, this study is among the first attempts to systematically explore correlations between HRCT imaging phenotypes and circulating biomarkers, contributing to a more integrated description of COVID-19 lung injury. These findings provide pathophysiological insight into the interplay between parenchymal and vascular lung involvement in severe COVID-19, and should be regarded as hypothesis-generating rather than clinically actionable. Within this framework, our results may inform future research aimed at refining the biological characterization of disease heterogeneity, identifying novel biomarkers, and generating hypotheses for targeted therapeutic strategies addressing both vascular and parenchymal components of viral lung disease. Larger, multi-centric prospective data, including other forms of ARDS, are warranted to validate and extend these observations and to clarify their potential clinical relevance in the context of personalized management strategies, ultimately leading to improved patient outcomes.

## Data Availability

The data that support the findings of this study are not openly available due to reasons of sensitivity and are available from the corresponding author upon reasonable request.

## Supplementary Materials

The following supporting information can be downloaded at: https://www.mdpi.com/article/10.3390/diagnostics16040587/s1, Table S1: Patients’ characteristics, clinical and laboratory parameters, referring to patients with low CT parenchymal score, high CT parenchymal score, and the total study population; Table S2: Patients’ characteristics, clinical and laboratory parameters, referring to patients with low CT vascular score and high CT vascular score.
