# Supplementary material for: Integrated Analysis of Parenchymal and Vascular HRCT Patterns with Circulating Biomarkers in Severe COVID-19 Pneumonia
Source: Diagnostics (Basel). 2026 Feb 15;16(4):587. doi: 10.3390/diagnostics16040587 (PMC12939422; doi:10.3390/diagnostics16040587)
Supplement: Supplementary file 1 [file diagnostics-16-00587-s001.zip › diagnostics-4074173-supplementary.pdf]

		Low Parenchymal Score					*High Parenchymal Score*					*Total population*				
		n	25th percentile	75th percentile	median	percentage	n	25th percentile	75th percentile	median	percentage	n	25th percentile	75th percentile	median	percentage
Age (years)		21	60,00	79,00	71,00		63	58,00	72,00	65,00		84	59,00	73,00	67,00	
Sex	Male	14				66,7%	46				73,0%	60				71,4%
	Female	7				33,3%	17				27,0%	24				28,6%
Height (m)		21	1,70	1,80	1,70		63	1,70	1,80	1,80		84	1,70	1,80	1,70	
BMI (kg/m²)		21	22,90	29,40	24,20		63	24,25	32,85	27,85		84	23,50	30,90	26,10	
WBC (×10³/µL)		21	6,30	11,60	9,15		63	6,30	11,60	7,85		84	6,30	11,60	8,30	
Neutrophils (×10³/µL)		21	4,40	9,60	7,85		63	4,30	9,80	6,10		84	4,40	9,70	6,65	
Lymphocytes (×10³/µL)		21	,50	1,00	,80		63	,60	1,10	,80		84	,60	1,05	,80	
Monocytes (×10³/µL)		21	,26	,80	,45		63	,27	,66	,51		84	,27	,80	,49	
Eosinophils (×10³/µL)		21	0,00	,06	,02		63	0,00	,13	,03		84	0,00	,10	,02	
Basophils (×10³/µL)		21	,01	,02	,02		63	,01	,03	,01		84	,01	,03	,01	
RBC (×10⁶/µL)		21	3,80	4,90	4,40		63	3,60	4,30	4,00		84	3,70	4,50	4,15	
HB (g/dL)		21	11,30	14,80	12,15		63	10,50	12,40	11,45		84	10,55	13,35	11,60	
Platelets (×10³/µL)		21	181,00	401,00	262,50		63	241,00	391,00	275,50		84	235,50	396,00	272,00	
Fibrinogen (mg/dL)		21	393	722	667		63	559	862	745		84	559	823	700	
D-dimer (ng/mL)		21	1,00	2,00	1,35		63	1,10	3,50	1,80		84	1,10	3,40	1,80	
Serum Creatinine (mg/dL)		21	,70	1,50	,85		63	,70	1,10	,90		84	,70	1,10	,90	
CRP (mg/L)		21	,90	15,40	9,10		63	7,00	21,00	11,80		84	5,20	19,40	11,05	
PCT (ng/mL)		21	,20	,90	,25		63	,20	,60	,30		84	,20	,60	,30	
ICU stay (days)		21	3,00	25,00	11,00		63	9,00	37,00	25,00		84	7,00	35,00	24,00	
hospitalization (days)		21	10,00	32,00	17,00		63	25,00	52,00	35,00		84	16,00	49,00	32,00	
sE-Selectin (ng/mL; t1)		21	14,73	28,42	23,63		63	19,53	49,67	27,49		84	18,48	41,45	26,33	
Angiopoietin 2 (ng/mL; t1)		21	1,75	3,11	2,45		63	1,58	4,42	2,76		84	1,68	4,02	2,68	
sVCAM-1 (ng/mL; t1)		21	653,58	1314,65	846,01		63	824,05	1866,44	1118,77		84	793,29	1643,63	1088,10	
sICAM-1 (ng/mL; t1)		21	201,24	993,35	416,93		63	165,25	720,82	275,42		84	177,83	775,93	353,17	
IFN-alpha2 (pg/mL; t1)		21	6,85	37,33	22,13		63	8,00	40,20	19,99		84	7,92	40,20	19,99	
IFN-gamma (pg/mL; t1)		21	3,20	5,49	4,01		63	3,20	12,28	4,21		84	3,20	11,62	4,08	
IL-1Ra (pg/mL; t1)		21	8,09	53,94	19,63		63	15,91	152,86	42,36		84	12,20	130,87	33,09	

IL-6 (pg/mL; t1)		21	3,20	34,44	3,94		63	3,20	146,21	28,08		84	3,20	119,06	18,12	
IL-10 (pg/mL; t1)		21	3,20	33,52	9,03		63	4,15	100,15	21,52		84	3,81	57,90	16,53	
IL-13(pg/mL; t1)		21	3,20	16,84	6,40		63	3,20	14,51	3,73		84	3,20	15,32	6,40	
s-RAGE (pg/mL; t1)		21	51,12	243,62	109,74		63	56,74	369,36	183,58		84	52,39	369,36	160,00	
Parenchymal score		21	1,00	1,00	1,00		63	2,00	3,00	3,00		84	1,50	3,00	2,00	
Vascular score		21	0,00	2,00	0,00		63	0,00	3,00	1,00		84	0,00	2,00	0,00	
Asthma	No	14				100,0%	32				100,0%	46				100,0%
	Yes	0				0,0%	0				0,0%	0				0,0%
COPD	No	13				92,9%	27				84,4%	40				87,0%
	Yes	1				7,1%	5				15,6%	6				13,0%
Dialysis	No	14				100,0%	31				96,9%	45				97,8%
	Yes	0				0,0%	1				3,1%	1				2,2%
Cancer	No	5				35,7%	18				56,3%	23				50,0%
	Current	1				7,1%	3				9,4%	4				8,7%
	Previous	8				57,1%	11				34,4%	19				41,3%
Transfer to ICU	No	11				78,6%	10				31,3%	21				45,7%
	Yes	3				21,4%	22				68,8%	25				54,3%
Respiratory support	Oxygen	7				36,8%	7				11,5%	14				17,5%
	HFNC	3				15,8%	10				16,4%	13				16,3%
	NIV	7				36,8%	20				32,8%	27				33,8%
	IOT	2				10,5%	24				39,3%	26				32,5%

Table S1: Patients' characteristics, clinical and laboratory parameters, referring to patients with low CT parenchymal score, high CT parenchymal score, and the total study population. Abbreviations: BMI: Body Mass Index; COPD: Chronic Obstructive Pulmonary Disease; CRP: C-Reactive Protein; HFNC: High-Flow Nasal Cannula; ICAM: Intercellular Adhesion Molecule; ICU: Intensive Care Unit; IFN: Interferon; IL: Interleukin; IOT: Orotracheal Intubation; NIV: Non-Invasive Ventilation; PCT: Procalcitonin; RAGE: Receptor for Advanced Glycation End Products; RBC: Red Blood Cells; sE-Selectin: Soluble E-Selectin; sVCAM: Soluble Vascular Cell Adhesion Molecule; VCAM: Vascular Cell Adhesion Molecule; WBC: White Blood Cells.

		Low Vascular Score					*High Vascular Score*				
		n	25th percentile	75th percentile	median	percentage	n	25th percentile	75th percentile	median	percentage
Age (years)		43	55,00	73,00	67,00		41	60,00	73,00	67,00	
Sex	Male	31				72,1%	29				70,7%
	Female	12				27,9%	12				29,3%
Height (m)		43	1,70	1,80	1,70		41	1,70	1,80	1,80	
BMI (kg/m²)		43	23,10	30,90	27,90		41	23,90	29,30	25,80	
WBC (×10³/μL)		43	6,00	9,90	7,85		41	6,50	12,60	8,60	
Neutrophils (×10³/μL)		43	4,40	8,10	6,30		41	5,00	10,80	7,20	
Lymphocytes (×10³/μL)		43	0,50	1,20	0,85		41	0,70	1,00	0,75	
Monocytes (×10³/μL)		43	0,21	0,90	0,55		41	0,29	0,66	0,49	
Eosinophils (×10³/μL)		43	0,02	0,09	0,05		41	0,00	0,10	0,02	
Basophils (×10³/μL)		43	0,01	0,02	0,02		41	0,01	0,03	0,01	
RBC (×10⁶/μL)		43	3,80	4,70	4,20		41	3,60	4,40	4,05	
HB (g/dL)		43	10,60	14,00	11,65		41	10,50	13,20	11,60	
Platelets (×10³/μL)		43	229,00	369,00	289,50		41	241,00	415,00	270,00	
Fibrinogen (mg/dL)		43	579	757	683		41	532	883	724	
D-dimer (ng/mL)		43	1,10	3,50	2,30		41	1,10	2,80	1,70	
Serum Creatinine (mg/dL)		43	0,70	1,10	0,85		41	0,70	1,10	0,90	
CRP (mg/L)		43	3,00	15,40	10,55		41	5,40	21,00	11,05	
PCT (ng/mL)		43	0,20	0,90	0,30		41	0,20	0,50	0,30	
ICU stay (days)		43	5,00	25,00	17,00		41	9,00	43,00	25,00	
hospitalization (days)		43	12,00	49,00	31,50		41	17,00	52,00	33,00	
sE-Selectin (ng/mL; t1)		43	17,76	34,95	24,94		41	19,24	53,63	26,33	
Angiopoietin 2 (ng/mL; t1)		43	1,45	3,16	2,32		41	2,10	5,36	2,85	
sVCAM-1 (ng/mL; t1)		43	742,28	1718,12	962,19		41	835,52	1612,22	1139,85	
sICAM-1 (ng/mL; t1)		43	182,17	640,83	305,34		41	190,75	993,35	397,40	
IFN-alpha2 (pg/mL; t1)		43	8,00	41,48	22,13		41	6,85	37,99	15,62	
IFN-gamma (pg/mL; t1)		43	3,20	12,28	5,04		41	3,20	5,64	3,20	
IL-1Ra (pg/mL; t1)		43	15,91	100,83	46,58		41	9,33	138,43	30,15	

IL-6 (pg/mL; t1)		43	3,20	45,60	12,91		41	3,20	149,22	34,17	
IL-10 (pg/mL; t1)		43	3,74	57,90	14,70		41	4,15	54,82	18,76	
IL-13(pg/mL; t1)		43	3,20	14,51	5,53		41	3,20	15,32	6,40	
s-RAGE (pg/mL; t1)		43	56,73	367,09	206,48		41	51,12	369,36	133,30	
Parenchymal score		43	1,00	3,00	2,00		41	2,00	4,00	3,00	
Vascular score		43	0,00	0,00	0,00		41	2,00	4,00	2,00	
Asthma	No	18				100,0%	28				100,0%
	Yes	0				0,0%	0				0,0%
COPD	No	16				88,9%	24				87,0%
	Yes	2				11,1%	4				13,0%
Dialysis	No	18				100,0%	28				100,0%
	Yes	0				0,0%	0				0,0%
Cancer	No	16				88,9%	27				93,5%
	Current	2				11,1%	1				6,5%
	Previous	18				100,0%	27				97,8%
Transfer to ICU	No	0				0,0%	1				2,2%
	Yes	2				11,1%	21				50,0%
Respiratory support	Oxygen	0				0,0%	4				8,7%
	HFNC	16				88,9%	3				41,3%
	NIV	9				50,0%	12				45,7%
	IOT	9				50,0%	16				54,3%

Table S2: Patients' characteristics, clinical and laboratory parameters, referring to patients with low CT vascular score and high CT vascular score. Abbreviations: BMI: Body Mass Index; COPD: Chronic Obstructive Pulmonary Disease; CRP: C-Reactive Protein; HFNC: High-Flow Nasal Cannula; ICAM: Intercellular Adhesion Molecule; ICU: Intensive Care Unit; IFN: Interferon; IL: Interleukin; IOT: Orotracheal Intubation; NIV: Non-Invasive Ventilation; PCT: Procalcitonin; RAGE: Receptor for Advanced Glycation End Products; RBC: Red Blood Cells; sE-Selectin: Soluble E-Selectin; sVCAM: Soluble Vascular Cell Adhesion Molecule; VCAM: Vascular Cell Adhesion Molecule; WBC: White Blood Cells.
